# Factors Associated With Delayed Diagnosis of Breast Cancer in Northeast Thailand

**DOI:** 10.2188/jea.JE20130090

**Published:** 2014-03-05

**Authors:** Amornsak Poum, Supannee Promthet, Stephen W Duffy, Donald Maxwell Parkin

**Affiliations:** 1 Graduate School, Khon Kaen University, Khon Kaen, Thailand; 2 Department of Epidemiology, Faculty of Public Health, Khon Kaen University, Khon Kaen, Thailand; 3 Centre for Cancer Prevention, Wolfson Institute for Preventive Medicine, Queen Mary University of London, London, United Kingdom

**Keywords:** delayed diagnosis, advanced stage, breast cancer, Thailand

## Abstract

**Background:**

We identified factors associated with delayed first consultation for breast symptoms (patient delay), delayed diagnosis after first consultation (doctor delay), and advanced pathologic stage at presentation among 180 women with breast cancer in Thailand.

**Methods:**

In this cross-sectional study 180 patients with invasive breast cancer were interviewed about potential risk factors and markers of delayed presentation. Patient delay was defined as time from onset of symptoms to first consultation with a health care provider, and doctor delay was defined as time from first consultation with a health care provider to diagnosis of breast cancer. Linear regression and logistic regression were used for the data analyses.

**Results:**

Among the 180 patients, 17% delayed seeking consultation for longer than 3 months, and 42% reported a doctor delay of longer than 3 months. In multivariate linear analysis, a significant increase in patient delay was associated with higher family income and smoking; factors associated with increased doctor delay were previous breast symptoms, self-treatment, and travel time to the hospital. In multiple logistic regression, doctor delay was related to age at first birth (*P* = 0.003), previous breast symptoms (*P* = 0.01), and number of consultations with a surgeon before diagnosis (*P* = 0.007). Regarding stage of breast cancer, there were significant associations with age at diagnosis (*P* for trend = 0.04), education (*P* for trend = 0.01), family income (*P* for trend = 0.02), time to referral (*P* = 0.01), and number of consultations with a surgeon before diagnosis (*P* < 0.01).

**Conclusions:**

Hospital referral from a health care provider was a major contributor to delayed diagnosis. Breast cancer awareness campaigns in Thailand should target individuals in low- and high-income groups, as well as practitioners.

## INTRODUCTION

Delayed presentation of breast cancer is a problem in developed and developing countries.^1^ To improve stage distribution, possible issues to address include public awareness of breast cancer and its symptoms, access to screening and diagnostic services, primary care awareness and referral time, and resources and practices at secondary and tertiary breast care services.^2^^,^^3^

Stage at presentation is more advanced in developing countries than in developed countries.^3^^,^^4^ Delayed presentation and more advanced stage at diagnosis were found to be associated with low socioeconomic status in developed and developing countries.^5^^–^^7^ In addition, a number of ethnic groups tended to present late with the disease.^8^^,^^9^ While breast cancer has traditionally been a major health problem in Western Europe, North America, and Australia, its incidence has been increasing in other regions, notably many Asian countries, including Thailand.^2^^,^^3^ Although basic health care services are currently free-of-charge through the national health insurance system in Thailand, different payment systems were in effect during the period of the present study. These included the “30 baht” scheme, which refers to the maximum charge for any health service visit. Hospital services may also be paid for by private health insurance. Normally, after consultation at a primary health care provider, referral for specialist treatment will depend on disease severity and the type of insurance cover. However, many patients seek health care by themselves (so called “self-treatment”), eg, by buying medicine from pharmacies, using alternative medicine, or going to a private clinic or private hospital. The extent of the delay between symptom onset and diagnosis therefore depends on several aspects of patient behavior and beliefs, as well as the physical and financial accessibility of appropriate primary and secondary health care services.

There is a lack of evidence with respect to breast cancer regarding the risk factors for delayed diagnosis and advanced stage in such settings. We therefore attempted to identify factors associated with delayed first consultation for breast symptoms (patient delay), delayed diagnosis after first consultation (doctor delay), and advanced pathologic stage at presentation in 180 women with breast cancer in Thailand.

## METHODS

Data collection was carried out during the period from May through December 2009 at 2 tertiary hospitals in Khon Kaen Province and 1 in Udontani Province, Thailand. In total, 190 women with newly diagnosed invasive breast cancer were eligible for the study. About 5% of these women declined to participate, and 180 participants were enrolled.

All participants were interviewed by a trained nurse within 3 months of diagnosis. Interviews required 45 to 60 minutes to complete and included information on potential breast cancer risk factors, including social, reproductive, and medical factors; knowledge and attitudes towards breast cancer; and health care practices. The Appendix lists the variables examined in the questionnaire. In addition, the timing of the diagnosis was examined by eliciting the dates of initial symptoms, first consultation with a health care provider (ie, nurse, physician doctor, or public health officer), first referral to hospital, and diagnosis of breast cancer. In addition, we used patient records to retrieve details on the cancers diagnosed, in particular, pathologic stage.

We defined patient delay as time from first reported symptoms to first consultation with a health provider, and doctor delay as time from first consultation with a health provider to diagnosis of breast cancer. Referral time (the period between first consultation with the health care provider to first referral to hospital) is a component of doctor delay. We expected referral time due to patient delay to be minimal because Thai patients are very highly motivated to accept and attend hospital appointments, due to the widely known extreme pressures on the health system and the potentially long waiting lists for appointments. Another factor is that Thais have a very high regard for medical practitioners and thus wish to comply with their busy schedules whenever possible.

Factors affecting patient and doctor delay were identified by 2 analyses: first, by linear regression of the delay in days on risk factors, knowledge, attitudes, and practices; second, by dichotomizing the delay to 3 months or less or longer than 3 months and performing logistic regression^10^ to identify factors associated with a delay of longer than 3 months, relative to a delay of 3 months or less. Finally, we defined stage 1 or 2 as early stage and stage 3 or 4 as advanced stage at diagnosis and used logistic regression to identify factors associated with advanced stage at diagnosis.

In all regression analyses, we first performed univariate analysis, assessing the influence of each factor in isolation. We then fitted all factors that were significant (*P* < 0.05) in univariate analysis in a multiple regression model, to identify those factors with the strongest independent effects on early and advanced stage. Statistical analysis was performed using STATA Version 10.^11^

This research was approved by the Khon Kaen University Ethics Committee for Human Research and adhered to the requirements of the Declaration of Helsinki and Good Clinical Practice Guidelines (ICH GCP), Reference No. HE511074.

## RESULTS

Median patient delay was 12 days, and median doctor delay was 21 days. Table 1 shows the basic characteristics of the study participants. Average age (SD) at diagnosis was 50 (11) years (range 25–83 years). Of the 180 patients, 58 (32%) had received a secondary school education or higher and 119 (66%) were employed or in business. Average monthly household income was 8852 baht (US $277). In total, 118 (66%) of the patients initially presented with a lump. Half of patients received free treatment, and one quarter were covered by health insurance schemes. Only 15 patients were fully self-paying. For most patients the first consultation was at a general hospital (in rural areas, a small district hospital) or private clinic (*n* = 32). Among the few patients (*n* = 19; 11%) who first attended a government primary health center, the first consultation was with a health worker or nurse rather than a doctor.

**Table 1.** Characteristics of the participants (*n* = 180)

Factors	Number	%
Age at diagnosis (years)		
<40	34	19
40–60	112	62
>60	34	19
Mean/SD, 50/11		
Education		
Primary school	122	68
Secondary school	29	16
Diploma	7	4
Graduate or higher	22	12
Employment status		
Not employed	61	34
Employed	119	66
Family income (baht/month)		
<10 000	146	81
10 000–20 000	23	13
>20 000	11	6
Mean/SD, 8852/11 812		
First symptom of breast cancer		
Lump/tumor	118	66
Change in nipple/breast	14	8
Bleeding/discharge	6	3
Dimpling	5	3
Lump under armpit	14	8
Painless	19	10
Rash	4	2
Health care provider first consulted		
Doctor	159	88
Health worker or nurse	21	12
First consultation		
Hospital or clinic	161	89
Health care center	19	11
Method of hospital payment		
Insurance	45	25
30 baht scheme	30	17
None (free of charge)	90	50
Self-payment	15	8
Stage (pathologic diagnosis)		
I	22	12
II	68	38
III	74	41
IV	16	9

Some patient variables were correlated. For example, family income was associated with educational level (*P* < 0.001), and the person conducting the first consultation (doctor, health worker, or nurse) was strongly associated with place of consultation (health center or hospital/clinic). However, family income was not related to patient employment status.

Table 2 shows the results of linear regression analysis of patient delay. In univariate analyses, significant increases in delay were associated with higher family income (*P* < 0.01), previous breast symptoms not pertaining to the current diagnosis (*P* = 0.02), and tobacco smoking (*P* < 0.01). In multiple regression analysis that included these 3 variables, income (*P* = 0.01) and smoking (*P* < 0.01) remained significant, while previous breast symptoms had a suggestive but nonsignificant effect (*P* = 0.08).

**Table 2.** Results of linear regression analyses of patient delay in breast cancer, by family income, previous breast symptoms, and smoking status

Factors	Number (%)	Mean (SD) delay, days	*P*-value	

			Univariate	Multivariate^a^
Family income (baht/month)			<0.01	0.01
<10 000	146 (81.1)	54.3 (147.7)		
10 000–20 000	23 (12.8)	192.6 (449.5)		
>20 000	11 (6.1)	186.6 (380.1)		
Previous breast symptoms			0.02	0.08
Yes	93 (51.7)	120.0 (310.1)		
No	87 (48.3)	36.9 (68.5)		
Smoking			<0.01	<0.01
Yes	5 (2.8)	371.4 (332.9)		
No	175 (97.2)	71.5 (223.3)		

^a^Adjusted for other 2 variables.

When patient delay was dichotomized (>3 vs ≤3 months; Table 3), higher family income (*P* = 0.04) and smoking (*P* = 0.02) were significantly associated with a long delay. Those who sought medical attention for a breast symptom on the basis of advice from family or friends were significantly more likely to have a delay (*P* = 0.02). In multivariate analysis that included these 3 variables, none of the variables were significant, but suggestive effects remained for income (*P* for trend = 0.1) and seeking medical care on the advice of family or friends (*P* = 0.10).

**Table 3.** Results of logistic regression analysis of patient delay in breast cancer, by family income, smoking, and recommending person

Factors	Patient delay		Crude OR	Adjusted^a^		
	
	≤3 months No. (%)	>3 months No. (%)		OR	95% CI	*P*-value
Family income (baht/month)						
<10 000	126 (84.0)	20 (66.7)	1	1		
10 000–20 000	16 (10.7)	7 (23.3)	2.75	2.83	1.01–7.95	0.04
>20 000	8 (5.3)	3 (10.0)	2.36	1.99	0.44–8.98	0.37
						*P*(trend) = 0.10
Smoking						
Yes	2 (1.3)	3 (10.0)	1	1		
No	148 (98.7)	27 (90.0)	0.12	0.15	0.02–1.03	0.06
Consultation recommendation						
Patient	111 (74.0)	16 (53.3)	1	1		
Relative or friend	39 (26.0)	14 (46.7)	2.49	2.00	0.85–4.68	0.10

^a^Adjusted for all variables in table.

Table 4 shows the results of linear regression for doctor delay in univariate analysis. Employed status (*P* = 0.01), previous breast symptoms (*P* < 0.01), self-treatment (*P* = 0.05), longer distance from home to hospital (*P* < 0.01), increased travel time from home to hospital (*P* = 0.01), and more advanced stage (*P* < 0.01) were associated with significantly greater delays. Multivariate analysis separately evaluated distance to hospital (Model 1) and travel time to hospital (Model 2), because of the strong collinearity between these variables (Table 4). Self-treatment (*P* ≤ 0.02), previous breast symptoms (*P* ≤ 0.02), and stage (*P* < 0.01) remained significant, and occupational status had a suggestive effect (*P* = 0.07–0.09). Distance from, and time to, hospital were both significant (when examined separately).

**Table 4.** Linear regression analysis of doctor delay in breast cancer, by various factors

Factors	Number (%)	Mean (SD) delay, days	*P*-value		

			Univariate	Multivariate^a^	Multivariate^b^
Employment status			0.01	0.07	0.09
Not employed	61 (33.9)	75.4 (69.1)			
Employed	119 (66.1)	120.6 (126.9)			
Previous breast symptoms			<0.01	0.02	0.01
Yes	93 (51.7)	127.9 (129.8)			
No	87 (48.3)	81.1 (85.1)			
Self-treatment			0.05	0.01	0.02
Yes	46 (25.6)	132.9 (138.1)			
No	134 (74.4)	95.8 (101.4)			
Distance from hospital (km)			<0.01	<0.01	
≤5	82 (45.6)	81.2 (69.3)			
>5	98 (54.4)	125.5 (136.0)			
Travel time to hospital (minutes)			0.01		0.01
≤60	78 (43.3)	80.7 (74.7)			
>60	102 (56.7)	124.1 (131.9)			
Stage (pathologic diagnosis)			<0.01	0.01	0.01
I	22 (12.2)	72.8 (44.2)			
II	68 (37.8)	93.9 (102.8)			
III	74 (41.1)	111.8 (106.7)			
IV	16 (8.9)	168.1 (198.7)			

^a^Adjusted for occupation, previous breast symptom, self-treatment, distance from hospital, stage from pathologic diagnosis.

^b^Adjusted for occupation, previous breast symptom, self-treatment, travel time to hospital, stage from pathologic diagnosis.

Table 5 shows the results of logistic regression for doctor delay. In univariate analysis, delay was significantly associated with higher parity (*P* < 0.05), early age at first birth (*P* < 0.01), previous breast symptoms (*P* < 0.01), first consultation at a general health care center (*P* < 0.05), first consultation with a doctor rather than another type of health care worker (*P* < 0.05), inconclusive initial diagnosis (*P* < 0.05), longer distance to hospital (*P* < 0.05), longer travel time to hospital (*P* < 0.01), and higher number of consultations with a surgeon before diagnosis (*P* < 0.01). In multivariate analysis, distance to hospital was excluded because of collinearity with travel time to hospital. The only remaining significant effects were age at first birth (*P* = 0.003), previous breast symptoms (*P* = 0.01), travel time to hospital (*P* = 0.01), and number of meetings with a surgeon before diagnosis (*P* = 0.007).

**Table 5.** Logistic regression analyses of doctor delay in breast cancer, by various factors

Factors	Doctor delay		Crude OR	Adjusted^a^		
	
	≤3 months No. (%)	>3 months No. (%)		OR	95% CI	*P*-value
Parity						
>2	44 (41.9)	39 (52.0)	1	1		
≤2	46 (43.8)	33 (44.0)	0.81	0.92	0.41–2.05	0.85
0	15 (14.3)	3 (4.0)	0.22^b^	N/A	N/A	N/A
Age at first birth (years)						
≤20	35 (33.3)	46 (61.3)	1	1		
>20	55 (52.4)	26 (34.7)	0.35^c^	0.29	0.13–0.65	0.003
Previous breast symptoms						
Yes	45 (42.9)	48 (64.0)	1	1		
No	60 (57.1)	27 (36.0)	0.42^c^	0.36	0.16–0.80	0.01
First consultation						
Health care center	99 (94.3)	62 (82.7)	1	1		
Hospital or clinic	6 (5.7)	13 (17.3)	3.45^b^	2.24	0.43–11.70	0.33
Health care provider first consulted						
Doctor	98 (93.3)	61 (81.3)	1	1		
Health worker or nurse	7 (6.7)	14 (18.7)	0.28^b^	1.39	0.26–7.21	0.69
Initial diagnosis						
Breast cancer	52 (49.5)	25 (33.3)	1	1		
Inconclusive	53 (50.5)	50 (66.7)	1.96^b^	1.69	0.78–3.66	0.17
Hospital payment method						
Insurance	33 (31.4)	12 (16.0)	1	1		
30 baht scheme	10 (9.5)	20 (26.7)	5.50	4.6	1.27–16.68	0.02
None (free of charge)	53 (50.5)	37 (49.3)	1.91	1.46	0.55–3.85	0.44
Self payment	9 (8.6)	6 (8.0)	1.83	1.42	0.32–6.24	0.64
Distance from hospital (km)						
≤50	55 (52.4)	27 (36.0)	1			
>50	50 (47.6)	48 (64.0)	1.95^b^			
Travelling time to hospital (minutes)						
≤60	54 (51.4)	24 (32.0)	1	1		
>60	51 (48.6)	51 (68.0)	2.25^c^	2.66	1.17–6.04	0.01
No. of consults with surgeon						
≤2	71 (67.7)	36 (48.0)	1	1		
>2	34 (32.3)	39 (52.0)	2.26	2.93	1.33–6.44	0.007

^a^Adjusted for all variables in table, except distance from hospital.

^b^*P* < 0.05, ^c^*P* < 0.01.

Table 6 shows the results of logistic regression for stage of breast cancer. In univariate analysis, late stage at diagnosis was significantly associated with older age (*P* for trend = 0.04), lower level of education (*P* for trend = 0.01), lower family income (*P* for trend = 0.02), postmenopausal status (*P* = 0.02), long referral time (*P* = 0.01), and lower number of consultations with a surgeon before diagnosis (*P* = 0.02). In multivariate analysis, the only remaining significant effects were age at diagnosis (*P* for trend = 0.04), increased time to referral from first health care provider to hospital (*P* = 0.01), and lower number of consultations with a surgeon before diagnosis (*P* < 0.01).

**Table 6.** Logistic regression analyses of staging in breast cancer, by various factors

Factors	Stage		Crude OR	Adjusted^a^		
	
	Stage 1/2 No. (%)	Stage 3/4 No. (%)		OR	95% CI	*P*-value
Age at diagnosis (years)						
<40	23 (25.6)	11 (12.2)	1	1		
40–60	49 (54.4)	63 (70.0)	2.68	2.13	0.82–5.54	
>60	18 (20.0)	16 (17.8)	1.85	1.25	0.37–4.17	
						*P*(trend) = 0.04
Education						
Primary school	54 (60.0)	68 (75.6)	1	1		
Secondary school	16 (17.8)	13 (14.4)	0.64	0.81	0.31–2.13	
Diploma	5 (5.5)	2 (2.2)	0.31	0.32	0.05–1.89	
Graduate or higher	15 (16.7)	7 (7.8)	0.37	0.62	0.18–2.16	
						*P*(trend) = 0.01
Family income (baht/month)						
<10 000	68 (75.6)	78 (86.7)	1	1		
10 000–20 000	13 (14.4)	10 (11.1)	0.67	0.70	0.24–2.05	
>20 000	9 (10.0)	2 (2.2)	0.20	0.28	0.04–1.74	
						*P*(trend) = 0.02
Menopause status						
Premenopause	45 (50.0)	30 (33.3)	1	1		
Postmenopause	45 (50.0)	60 (66.7)	2.00^b^	1.62	0.79–3.34	0.18
Time to referral (days)						
≤50	74 (82.2)	60 (66.7)	1	1		
>50	16 (17.8)	30 (33.3)	2.31^b^	2.56^b^	1.18–5.55	0.01
No. of consults with surgeon						
≤2	46 (51.1)	61 (67.8)	1	1		
>2	44 (48.9)	29 (32.2)	0.49^b^	0.41^c^	0.21–0.80	<0.01

^a^Adjusted for all variables in table.

^b^*P* < 0.05, ^c^*P* < 0.01.

## DISCUSSION

Although the 180 participants enrolled in the study represented more than 90% of the total number of eligible patients admitted to the 3 study hospitals during the enrollment period, they were somewhat younger (mean age = 50) than breast cancer cases occurring in the whole population, based on cancer registrations during the same period (mean age = 51). In addition the stage distribution of participants appeared to be slightly more favorable.^12^ The possibility of selection bias must therefore be considered.

We found that 17% of patients reported a delay of longer than 3 months, which is comparable with delays observed in Germany,^13^ the United Kingdom,^14^ and Colombia.^15^ Greater patient delay (ie, time from symptoms to first consultation with a healthcare provider) was associated with a history of previous breast symptoms, smoking, and unexpectedly, higher family income. Although we expected smoking to correlate with other high-risk health behaviors, we found no precedent in the literature for any of these 3 factors. Indeed, lower income was previously found to be associated with greater patient delay.^6^ Although it is possible that employment is a barrier to early consultation for symptoms, this has not been observed in other studies,^16^ and employment status was not associated with higher income in our case series.

Factors associated with greater doctor delay (time from first consultation with a healthcare provider to diagnosis of breast cancer) in multivariate analysis were previous breast symptoms, self-treatment, distance or travel time to hospital, younger age at first birth, and increased number of consultations with a surgeon before diagnosis. Again, these results were not replicated in the literature, although increased time to referral could be regarded as self-evident. The main predictors of doctor delay reported in previous research mainly pertain to the diagnostic process and include non-lump symptoms and false-negative or inappropriate investigations.^6^^,^^17^ Practitioner delay was also found to be associated with younger patient age and patient ethnic origin.^17^

We had no information on the number of health care practitioners who were first consulted by participants; nevertheless, given the geographic dispersal of the patients (12 different provinces) it is probable that few practitioners saw more than 1 participant. Doctor delay therefore varies considerably by practitioner. However, except for type of health worker (doctor, nurse, health worker), we have no information on the characteristics that might have influenced such delay.

Factors associated with later stage (3 or 4) at diagnosis in multivariate analysis were older age, lower level of education, lower family income, greater time to referral, and number of consultations with a surgeon before diagnosis. In univariate analysis, some of the results were similar, eg, late stage was associated with lower educational status and older age.^8^ This suggests that these factors influence variables related to the diagnostic process, such as referral time and number of surgical consultations, and thus their influence on stage at presentation is mediated through factors related to the diagnostic process. While a large number of surgical consultations was, surprisingly, not associated with longer doctor delay—and longer doctor delay was associated with late stage diagnosis—a stage 1 diagnosis was more strongly associated than a later-stage diagnosis with a greater number of consultations. This apparent paradox can be explained by the fact that although an early-stage disease is more difficult to diagnose and requires a larger number of visits for diagnostic investigations, these visits to confirm a suspected diagnosis occur over a short period, thereby resulting in minimal extra doctor delay.

While higher income was significantly associated with increased patient delay in univariate analysis, it was nevertheless also associated with early stage at diagnosis. One possible explanation for this is that since people on higher incomes were more highly educated, they had a better understanding of what in retrospect may have been a very early sign of breast cancer. This raises the possibility of a major problem in determining the beginning of patient delay when it is defined in terms of retrospective reports regarding when cancer symptoms were first noticed by a patient who is later discovered to have the disease. This is not simply an issue of recall accuracy but may be more accurately construed as an issue of post-hoc interpretation of symptoms.^18^

The median patient and doctor delays in our study (12 and 21 days, respectively) were similar to those reported in New Zealand and the United Kingdom.^19^^,^^20^

In conclusion, factors associated with late-stage breast cancer in Thailand were not substantially different from those one would expect from results elsewhere. However, factors associated with reported delay in breast cancer diagnosis differed from those observed in developed countries. Breast cancer awareness campaigns in Thailand should target low- and high-income individuals. In addition, practitioners, especially those in primary care, should be reminded of the importance of prompt referral.

## APPENDIX

Variables examined in the questionnaire were age, race, religion, marital status, height, weight, education, family income, occupation, menstruation, menopausal, parity, age at first birth, breast feeding, abortion history, contraception, smoking, alcohol drinking, family history of breast cancer, breast self-examination, previous breast symptoms, first consultation, type of health care provider consultation, self-treatment, health-seeking behavior, satisfaction, referral system, diagnostic history, timing, cost and distance of traveling, health insurance, first symptoms of breast cancer, pathologic type, pathologic stage, consultations with surgeon, tumor size, morphology, metastasis, treatment, knowledge of breast cancer, perception of breast cancer symptoms, severity, problems, and treatment.
